# Academic Failure and Child-to-Parent Violence: Family Protective Factors

**DOI:** 10.3389/fpsyg.2016.01538

**Published:** 2016-10-07

**Authors:** Izaskun Ibabe

**Affiliations:** Social Psychology and Behavioral Sciences Methodology, University of the Basque CountryDonostia-San Sebastián, Spain

**Keywords:** academic failure, academic achievement, school adjustment, family environment, gender differences, child-to-parent violence

## Abstract

A reduction in academic achievement over the course of adolescence has been observed. School failure is characterized by difficulties to teaching school goals. A variety of other behavioral problems are often associated with school failure. Child-to-parent violence has been associated with different school problems. The main objective of current study was to examine the contribution of family variables (parental education level, family cohesion, and positive family discipline) on academic failure and child-to-parent violence of adolescents from a community sample. Moreover, a goal was to explore if academic failure was a valid predictor of child-to-parent violence. To this end, it has been developed a comprehensive statistical model through Structural Equation Modeling (SEM). Participants were 584 children from eight secondary schools in the Basque Country (Spain) and aged between 12 and 18. Among other scales Conflict Tactics Scale and Family Environment Scale were administrated for measuring child-to-parent violence and family cohesion environment, respectively. The structural model revealed that parental education level is a relevant protective factor against academic failure. Positive family discipline (inductive discipline, supervision, and penalty) show a significant association with child-to-parent violence and academic failure. Disciplinary practices could be more efficient to prevent child-to-parent violence or school failure if children perceive a positive environment in their home. However, these findings could be explained by inverse causality, because some parents respond to child-to-parent violence or academic failure with disciplinary strategies. School failure had indirect effects on child-to-parent violence through family cohesion. For all that, education policies should focus on parental education courses for disadvantaged families in order to generate appropriate learning environments at home and to foster improvement of parent-child relationships.

## Introduction

School failure refers to students' difficulties for fulfilled teaching goals (fully or partially; Kalogridi, 1995), which can in extreme cases lead to their dropping out of school. Academic failure implies both poor academic performance and school maladjustment. It implies negative effects on social cohesion and mobility, and involves extra expenses on community budgets as a result of, for example, more public health problems, lack of social support, or criminality (OECD, the Organisation for Economic Co-operation Development, 2012). Thus, high academic failure and dropout rates remain significant issues in some countries. In the United States about 25% of public school students fail to earn a diploma (Stillwell, 2009). Rates of school failure in Spanish students are above the other European student and OECD countries average (Fernández et al., 2010). In Spain during the school year 2008/2009 the number of school children who not achieved a Certificate in Compulsory Secondary Education was 26%, and the goal for school dropout in 2020 is 15% (Organisation for Economic Co-operation Development, 2011). A decrease in academic achievement during adolescence has been found in previous research (e.g., Barber and Olsen, 2004; Hernando et al., 2012), while in many countries has been increased the attention to the underachievement of boys in comparison with girls (Jackson, 1998; Van Houtte, 2004). Van Houtte (2004) found that boys' culture is less study-oriented than girls, and that this difference could be the explanation of gender differences in school achievement, at least in secondary school. However, Harris (1998) explained this gender difference as based on the process of development during puberty, whereby girls begin to be disciplined and to take care in the planning and execution of their work earlier than boys.

Theoretical models of school performance developed in recent years have focused on students' personal characteristics (specific skills, motivation, or learning strategies; e.g., Loe and Feldman, 2007; Niepel et al., 2014), family variables (demographics, affective relationships, parenting styles, family educational involvement; Marchant et al., 2001; Sibley and Dearing, 2014), and school variables (school environment and education quality; Marchant et al., 2001; Organisation for Economic Co-operation Development, 2012). The present study is focused on family variables of school failure. The individual influences of family-related factors on students' achievement are well-documented in the literature: student's achievement is related to parenting style and parental involvement (e.g., Paulson, 1994; Sibley and Dearing, 2014). But recent studies have focused on how various risk factors come together to produce negative outcomes (Lucio et al., 2012). Family variables can be classified in demographic (socioeconomic level, parents' educational level and family structure) and dynamic (family environment, parenting style and family educational involvement).

### Socio-demographics family variables

Extensive research in the sociology of education has found a strong support for a positive association between family socio-economic status and academic achievement (Sirin, 2005; Caro et al., 2009). There is no strong agreement on the conceptual of socio-economic status, but it is generally operationalized through measures such as parents' education, parental occupational prestige, and family income (Hauser, 1994). It has been observed that poor parental care with serious deprivation of children's needs tends to yield poor academic performance (Osonwa et al., 2013). On the other hand, according to Caro et al.'s study (2009), academic performance among students from varying socio-economic backgrounds is similar during primary school. However, from the middle-school years to the beginning of high school, the gap is widening.

Students from advantaged socio-economic families are exposed to a diverse learning environment because the parents are more involved in their education; hence, their learning outcomes tend to be better. In any case, scholars from low-level socio-economic condition are twice as likely to present low achievement (Organisation for Economic Co-operation Development, 2012). In addition, it should be pointed out that today, many children living in disadvantaged families are from minorities or have an immigrant background (Heckman, 2011).

According to Li-Grining (2007), the problem of low school performance begins with parents' lack of education and a poor understanding of children's needs. In Spain, children of parents who completed only compulsory education (to age 16) constitute the majority of school failure cases (74% in the case of father's education; 71% in that of mother's education), according to studies with secondary education pupils (Fernández et al., 2010). Even so, the positive effects of parental education on school success of children might not be significant until parental education reaches at least high school diploma (Jensen, 2007). In any case, it seems obvious that higher levels of parents' education will result in greater involvement in their children's education, and this will promote school completion and success (Unger et al., 2000; Mapp, 2004). Epstein (2011) indicated some subtypes of parental participation in children's education, such as involvement in children's homework, high parental expectations or extracurricular activities with achievement outcomes. However, despite the extensive literature, particularly in relation to elementary and middle-school contexts, findings about the effects of family educational involvement on high-school students' outcomes are inconclusive (Strayhorn, 2010). What emerges, though, is that parenting style may have meditational effect in the relationship between parental involvement and academic achievement (Blondal and Adalbjarnardottir, 2009).

In recent years, research linking family structure and children's educational outcomes has done a great deal to elucidate how family disparities can create educational inequalities (Crosnoe and Wildsmith, 2011). Children living in a nuclear family achieve more academically than those living in other types of family structure (single-parent or blended family; Fernández et al., 2010; Córdoba et al., 2011). This finding was supported in Zill's (1996) review of research results from extensive longitudinal data: students from intact nuclear families showed better academic performance than students from other type of family. It seems that children benefit from family stability for emotional and psychological development.

### Dynamic family variables

Positive family environment (parents-children affective cohesion, parental support, parental monitoring, confidence and openness, and empathic family communication) has been positively related to children's better behavioral and psychological adjustment (Moreno et al., 2009; Jaureguizar and Ibabe, 2012). Furthermore, indirect effects of family cohesion on academic performance were found through parental involvement in school activities for children (Unger et al., 2000) and through children's academic self-concept (Rodríguez-Fernández et al., 2012). In childhood parents have a major influence on the school performance of their children. However, Spera (2005) indicated that in adolescence findings of previous studies are not consistent. Given an increased need for autonomy, adolescents could respond negatively to high levels of parental involvement.

On the other hand, the relation between family environment and academic performance can be considered bidirectional, since a positive family environment promotes good academic achievement, while family climate is often impaired by school failure (Hernando et al., 2012). Research on parenting has ignored the bidirectional interactive process in parent-child relationship (see Collins et al., 2000).

The complex process of socialization of children by parents includes both discipline and supervision from childhood to adulthood. The purpose of socialization is to promote and prevent certain behaviors in the children. Within the family context, children gradually internalize social standards and expectations, a process that facilitates greater self-regulation skills and responsibility for their own behaviors (Halpenny et al., 2010) and means that when they are adolescents they will need fewer family discipline strategies than in previous periods. Nevertheless, there is little research on the relationships between strategies of positive discipline or partly-punitive discipline and school achievement. Weiss and Schwarz (1996) found that academic aptitude and achievement results of college students from nondirective families (parents who showed low directive control, low assertive control and midhigh, or high supportiveness), excelled scholastically.

### Child-to-parent violence

Child-to-parent violence has been associated with different school problems as school maladjustment (Ibabe, 2015), learning difficulties and disruptive behavior (Ibabe and Jaureguizar, 2010), less student involvement and less task orientation (Ibabe et al., 2013). In general, juveniles who abused their parents compared to other young offenders besides external symptomatology showed internal symptomatology (Ibabe et al., 2014).

In the current study child-to-parent violence (CPV) is defined as violent behavior by adolescent children toward their parents which includes physical and psychological violence with zero tolerance criterion. Abuse of parents by their children has been a hidden family problem, but the last decade it was displayed. One of the peculiarities of CPV is that parents are seeking protection from their children when they have socially and economically more power, and in some cases they are stronger physically.

Taking into account the Gallagher (2008)'s review, CPV is not related to socio-economic (SES) status or is more common in families with higher SES. In general, children from socially disadvantaged families are found to consistently be more aggressive (Hill and Maughan, 2001), and low income is associated with higher rates of intimate partner violence (Hotaling and Sugarman, 1986). Why would children in families with higher socio-economic status be more likely to be violent to parents? It is possible that children of better educated parents to utilize mental health services when they have behavior problems of their children increasing the prevalence rates of CPV (Goodman et al., 1997).

Adolescents from traditional families showed more violent behavior than single-mother families with mother (Kennair and Mellor, 2007), blended families other type of families (Ibabe, 2014). Single-parent families and blended families are more vulnerable than traditional families, by possible conflict with her former partner or adjustment to new family members. All this means that in these families have a higher level of distress and limited resources to address the adolescent stage of the child.

Generally, family relationships characterized by support, warmth, communication, and autonomy are key for promoting appropriate development in adolescents (Oliva et al., 2008). So that, the positive parent-child relationships is considered as a protective factor of adolescents' verbal or physical abuse of their parents (Estévez and Navarro, 2009; Ibabe, 2015). Similarly, there are several evidences that support the relationship between victimization from parents toward children and violence from children toward parents taking into account community samples (e.g., Ibabe and Jaureguizar, 2011; Gámez-Guadix and Calvete, 2012) and offenders samples (child-to-parent offenders and other offenders; Contreras and Cano, 2016). Child-to-parent violence was strongly associated with the lack of emotional support (Calvete et al., 2014a), as well as with parents with unrealistic expectations, or deficit in communication skills (Paulson et al., 1990; Kennedy et al., 2010).

There are empirical evidences that poor parental discipline and supervision are a relevant risk factor for the antisocial behaviors in adolescence (Loeber et al., 1993; Yoshikawa, 1994). Although some authors (e.g., Beyers and Goossens, 1999; Estévez and Navarro, 2009) noted that parental discipline based on markedly permissive or authoritarian control is linked with child-to-parent violence or psychological maladjustment, there are not consistent empiric evidences for violent behavior toward parents. Family discipline strategies have been classified as power-assertive and inductive. Power-assertive disciplinary methods involve following a child's inappropriate behavior with a negative consequence (smacking, threats, or deprivation of privileges) without explanation or justification. Inductive discipline involves setting limits, setting up logical consequences, reasoning, and explaining (Holden, 2002). It has been found that parents of adolescents who perpetrated CPV made fewer attempts to make sure there were consequences for inappropriate behavior and exerted less supervision (Calvete et al., 2014b). Surprisingly, Ibabe and Bentler (2015) found that supervision and penalty (medium-level power-assertive discipline) were linked to more violent behavior of adolescents. At the same time, inductive discipline was not associated with less violence against parents. These findings could be due to some parents respond to CPV with coercive strategies.

They are noteworthy potential bidirectional effects between family environment and child-to-parent violence. Research on child development indicates bidirectional effects between parent–child relationships and child temperament (e.g., Chess and Thomas, 1996; DeHart et al., 2004). Additionally, children with difficult temperaments (i.e., with externalizing symptoms) are more vulnerable to inappropriate discipline than children with relatively easy temperaments (Van Zeijl et al., 2007). In any study was found that discipline strategies administrated inconsistently or different parenting styles (Maccoby and Martin, 1983; Baumrind, 1991) applied by father and mother can be related to violent behavior toward parents (e.g., Calvete et al., 2014a). It is remarkable that there is little research on acceptable discipline strategies such as inductive discipline, supervision, or penalty, as protective factors of adolescents' violent behavior toward their parents or academic failure.

### Objectives and hypothesis

The main goal of this study was to analyze the contribution of family socio-demographic and family dynamic variables (family cohesion and positive family discipline) on two indicators of adolescent maladjustment (academic failure and child-to-parent violence) from community sample. Moreover, other goal was to explore the relationship between school failure and child-to-parent violence. To this end, it has been developed a comprehensive statistical model of family protective factors through Structural Equation Modeling (SEM).

The hypotheses were as follows:

- Parents' education and family cohesion will have some direct effects on academic failure. This hypothesis is based on studies indicating a lower level of academic failure when parents have a higher level of education (Li-Grining, 2007; Caro et al., 2009; Fernández et al., 2010), or when family environment is positive (Unger et al., 2000).
- Positive family discipline strategies will be associated with academic failure and child-to-parent violence. This hypothesis was based on Ibabe and Bentler (2015)'s finding: penalty and supervision were positively related to violent behavior of adolescent against parents. The explanations could be in an inverse causality, that parents may more frequently apply discipline strategies when children show child-to-parent violence or have low academic achievement.
- Children who live in nuclear families will present lower levels of academic failure and child-to-parent violence than those living in other types of family structure (single-parent families, step-families). This hypothesis is consistent with the previous findings on academic achievement (e.g., Zill, 1996; Córdoba et al., 2011) and child-to-parent violence (Kennair and Mellor, 2007). The decline in children's psychosocial well-being after parental separation could explain their lower academic performance (Potter, 2010) and violent behavior against parents.
- School failure will have direct effects on child-to-parent violence and indirect effects through family cohesion. In previous studies school maladjustment has been associated with child-to-parent violence (Ibabe et al., 2013; Ibabe, 2014), both variables could be indicators of psychological distress in adolescents. Taking into account that we will study a community sample, school failure could be a source of considerable family tension which might interfere with satisfactory relations between adolescents and parents (Hurrelmann et al., 1988). In several previous studies low family cohesion has been found as an important risk factor of child-to-parent violent (e.g., Ibabe et al., 2013).

## Method

### Participants

A total of 584 adolescents participated in the study from eight secondary schools in the Basque Country (Spain) of both sexes (48% boys), and aged 12–18 years (*M* = 14.55; *SD* = 1.53). Forty-three percent of the participants were attended state (public) schools and the rest were private schools. Seventy-five percent lived in nuclear families, 14% in single-mother families, 7% in step-families, and 4% in extended families or other types. Forty-seven percent of the participants had passed all their subjects in the previous term. The distribution of sample by age and sex is uniform, χ^2^_(**N** = 528, 6)_ = 5.89, *p* = 0.44 (see Table 1).

**Table 1:** **Distribution of the sample Sex × Age**.

		**Sex**		**Total**
		**Boy**	**Girl**	
Age	12	26	20	46
	13	53	49	102
	14	56	66	122
	15	49	57	106
	16	48	45	93
	17	15	28	43
	18	7	9	16
Total		254	274	528

### Instruments

#### Socio-demographic data

A questionnaire was applied to assess socio-demographic variables of the children. Among the characteristics measured were sex, age, family structure, parental education level (none, only compulsory education –ESO-, further education/job training or university), parents' occupation, and country of origin.

#### Academic failure

This was defined as the extent to which the child had failed to attain the minimum goals set by the school at every level of education, together with lack of learning motivation. Academic performance was measured through the number of subjects the participant had failed. The question about number of failed subjects in the previous term had 4 answer options (0 fail, 1–4 fails, 5–10 fails, 10 or more fails). As regards lack of motivation for school work, participants were required to indicate their interest in their studies on a Likert scale (1 = Very low; 4 = Very high). In this study alpha coefficient of the scale was 0.63. This coefficient value was below recommended value and it could be questionable. However, it is not due to the absence of correlation between items (inter-item correlation *r* = 0.47) but the relatively small number of items of the scale. Spearman-Brown prophecy formula (for estimating the increased reliability expected to result from increase in scale length) indicated an adequate reliability coefficient for the scale, if it would have additional two parallel items (α = 0.77). In attempting to increase the coefficient alpha of a scale, the quality of items may be more important than the quantity of items (Netemeyer, 2001). The principal components analysis yielded a one-factor structure with an eigenvalue greater than 1 (1.46), and this factor accounted for 73% of the total variation.

#### Family environment

Three subscales (cohesion, conflict, and organization) of the Family Environmental Scale (FES; Moos and Moos, 1981; Spanish version by TEA Ediciones, 1984) were applied. Each subscale contains 9 items (e.g., “In my family we really help and support each other”) with true/false response format. Cohesion is defined as the degree of commitment and support family members provide for each other. In this study cohesion showed an acceptable internal consistency (α = 0.76). However, taking into account that conflict (α = 0.61) and organization (α = 0.52) subscales are not reliable (alpha < 0.70; Nunnally, 1978), these measures of family environment were discarded of all data analyses.

#### Family discipline

Family discipline strategies were measured by the Dimensions of Discipline Inventory (DDI-C; Straus and Fauchier, 2007; Spanish adaptation by Calvete et al., 2010). This inventory includes 26 items in order to assess family discipline from children point of view in their relationship with their father and mother (e.g., “How often do your parents give you extra chores?”). Although the inventory contents four general dimensions, in this study were applied three: *Penalty* (deprivation of privileges and restorative behavior), *Supervision* (ignoring misbehavior and monitoring) and *Inductive discipline* (diversion, explanation, and reward). Its items describe different situations related to family life and upbringing, which children are required to answer on a 5-point Likert-type scale (0 = Never; 4 = Almost always). In this study the internal consistency for three general dimensions varied from 0.82 (Inductive discipline) to 0.77 (Supervision).

#### Child-to-parent violence

Conflict Tactics Scale Child-Parents (CTS1; Straus et al., 1998). This instrument is composed of 13 items (e.g., Insult or threaten my father/mother) and includes three subscales: psychological violence, mild physical violence, and serious physical violence. Children had to answer taking into account the last year and using a scale with 5-point Likert-type scale (0 = Never; 4 = Almost always). Reliability results for this study are acceptable (serious physical violence α = 0.83, mild physical violence α = 0.79, psychological violence α = 0.85).

### Procedure

The sample of adolescents was obtained by means of cluster sampling from all secondary schools in the Basque Country (Spain). Firstly, schools were randomly selected, and then they had to confirm their availability and the willingness of their staff to collaborate in the research. Two schools denied their participation and were replaced by others with similar characteristics. After that into each selected school some classrooms were chosen taking into account the linguistic model (monolingual vs. bilingual) and the education level of participants, in order to get a balanced and representative sample. Head teachers of each school were informed about the objectives of the study in a 1-h presentation. A letter was sent to the parents about research project, after they had to inform whether or not they agreed to their children participate in the study. Students were informed about the confidentiality and anonymity of their answers. Before students filled out the questionnaire, the instructions for each scale were explained carefully. The questionnaires were administered during normal class time in 1-h sessions. Data collection was conducted during 2011, and administration time for the instruments was approximately 45 min. Initially, the 5% of children couldn't join in the study because their parents didn't give their consent to participate. After collecting data, the 4% of participants were discarded of the sample by errors or omissions in its answers to the tests or were outside of age range (12–18 years old).

### Data analysis

Univariate data analyses were carried out using PASW Statistics version 20. The first of these analyses included percentages corresponding to the socio-demographic characteristics of the sample and a matrix of correlation between academic failure and family variables (see Table 2 in which 12 observed variables were included with their means and standard deviations). Spearman rank correlation was used to measure the degree of association between number of failed subjects and school hypo-motivation with the rest of variables, being ordinal variables. Moreover, point-biserial correlation coefficient was applied when these variables were correlated with one dichotomous variable (immigrant or nuclear family).

**Table 2:** **Means, standard deviations and correlations between academic failure, child-to-parent violence, and family context variables**.

	***M/%***	***DT***	**1**	**2**	**3**	**4**	**5**	**6**	**7**	**8**	**9**	**10**	**11**
**ACADEMIC FAILURE**													
1. Number of subjects failed	1.68	0.74	–										
2. Hypo-motivation	2.29	0.88	0.47^**^	–									
**CHILD-TO-PARENT VIOLENCE**													
3. Physical violence	0.27	1.13	0.15^*^	0.12^**^	–								
4. Psychological violence	4.25	3.63	0.02	0.09^*^	0.22^**^	–							
**PARENTAL EDUCATION**													
5. Father's educational level	1.88	0.86	−0.27^**^	−0.18^**^	−0.07	−0.01	–						
6. Mother's educational level	1.93	0.89	−0.24^**^	−0.17^**^	−0.06	0.01	0.58^**^	–					
**POSITIVE FAMILY ENVIRONMENT**													
7. Family cohesion	6.74	2.22	−0.19^**^	0.18^**^	−0.24^**^	−0.41^**^	0.12^**^	0.13^**^	–				
**POSITIVE FAMILY DISCIPLINE**													
8. Penalty	0.85	0.69	0.13^*^	−0.01	0.11^*^	0.27^**^	0.05	−0.03	−0.06	–			
9. Parental supervision	0.65	0.65	0.19^**^	0.13^**^	0.17^**^	0.42^**^	0.02	−0.01	−0.23^**^	0.56^**^	–		
10. Inductive discipline	1.39	0.67	−0.05	−0.02	−0.05	0.21^**^	0.07	0.06	0.11^**^	0.53^**^	0.39^**^	–	
**DEMOGRAPHICS**													
11. Immigrant (Yes, No)	23%	–	0.27^**^	0.04	0.19^**^	0.01	−0.15^**^	−0.14^**^	−0.11^**^	0.15^**^	0.16^**^	−0.01	–
12. Nuclear family (Yes, No)	75%	–	−0.19^**^	−0.07	−0.18^**^	−0.11^*^	0.14^**^	0.08	0.19^**^	0.01	−0.07	0.09	0.34^**^^a^

** Correlation is significant p < 0.01;

* *p < 0.05*.

a *Contingency coefficient because two variables are qualitative*.

The adequacy of the proposed model was assessed using EQS 6.1 Structural Equations Program. Confirmatory factor analysis (CFA) assessed the adequacy of the hypothesized measurement model which included four latent factors and one observed variable (family cohesion). The first-order latent variables included in the CFA were: Parental Education Level (indicators: father's educational level and mother's educational level), Positive Family Discipline (indicators: penalty, supervision and inductive), Academic Failure (indicators: number of failed subjects and hypo-motivation), and Child-to-Parent Violence (indicators: psychological violence and physical violence).

Next, a structural model posited family cohesion with direct effects on Family Discipline, Academic Failure, and Child-to-Parent Violence. It was indicated direct and indirect effects of Parental Education on Academic Failure through family cohesion. Moreover, the model included two bidirectional relations: Positive Family Discipline with Academic Failure and Child-to-Parent Violence. Finally, in this model was indicated an association between Academic Failure and Child-to-Parent Violence.

The Yuan-Bentler scaled chi-square (χ^2^) (Yuan and Bentler, 2000) was calculated and the practical fit indexes (*IFI, CFI*, and *NNFI*) above 0.90 or higher were considered an indication of acceptable fit (Bentler, 2006). The *RMSEA* index values 0.01, 0.05, and 0.08 were interpreted as excellent, good, and mediocre fit, respectively (MacCallum et al., 1996).

Of the total participants 74% had complete data (*n* = 433) and the rest had at least one missing value. In total there were 36 patterns of missing values. Full-information maximum likelihood estimation method for missing data was carried out (e.g., Arbuckle, 1996; Jamshidian and Bentler, 1998). While the maximum likelihood estimates were accepted, since the Yuan et al. (2004) normalized coefficient of kurtosis (49.41) indicated a lack of normal distribution, the Yuan and Bentler (2000) robust methodology was used. All the observed variables had skewness and kurtosis coefficients below 1.8, except physical child-to-parent violence (skewness = 6.71 and kurtosis = 56.41). It is assumed that absolute values less than 1 indicate non-normality, and values between 1 and 2.3 indicate moderate non-normality (Lei and Lomax, 2005). Fit indexes based on robust statistics will be reported.

## Results

Forty-seven percent of participants had passed all their courses in the prior term, 41% had failed between 1 and 4 courses and 12% had failed 5 or more. As regards learning motivation, 8% of students reported low or very low interest in their studies, while 20% reported high or very high interest.

### Relation between academic failure, child-to-parent violence and family characteristics

In order to explore the relationship between academic failure and variables associated with family context, a correlation matrix was drawn up (see Table 2). On the one hand, it is found that academic failure (number of failed subjects and school hypo-motivation) is related to father's educational level (*r* = −0.27 and *r* = −0.18), mother's educational level (*r* = –0.24 and *r* = −0.17), family cohesion (*r* = −0.19 and *r* = −0.18), and degree of parental supervision (*r* = 0.19 and *r* = 0.13). On the other hand, number of failed subjects is associated with being an immigrant (*r* = 0.27) and living in a type of family that is not nuclear (single-parent family, step family, extended family, or other type; *r* = −0.19). On the other hand, child-to-parent violence (physical and psychological) was inversely associated with cohesion (*r* = −0.24 and *r* = −0.41). However, psychological child-to-parent violence was related to more penalty (*r* = 0.27), supervision (*r* = 0.42), and inductive discipline (*r* = 0.21).

In some complementary analysis on the relationship socio-economic level and academic failure, it was found that the number of failed subjects was associated with lower professional category of parents (father *r* = −0.21, *p* < 0.001; mother *r* = −0.27, *p* < 0.001), and parents' unemployment (father *r* = −0.12, *p* < 0.01; mother *r* = −0.18, *p* < 0.01). However, school hypo-motivation was related only to mother's unemployment (*r* = −0.12, *p* < 0.01). In general, these correlations were lower those found for parental education.

### Confirmatory factor analysis

A confirmatory factor analysis (CFA) assessed the adequacy of the hypothesized measurement model and the associations among the latent variables and one observed variable, *Y-B ML* χ^2^_(27, **N** = 584)_ = 102.77, *CFI* = 0.94, *NNFI* = 0.89, *IFI* = 0.94, *RMSEA* = 0.063. After adding one correlated errors path (family cohesion and inductive discipline, *r* = 0.22, *p* < 0.001), fit indexes for the CFA were all acceptable. *Y-B ML* χ^2^_(25, **N** = 584)_ = 81.46, *CFI* = 0.96, *NNFI* = 0.92, *IFI* = 0.96, *RMSEA* = 0.054. All factor loadings were highly significant (*p* < 0.001). Table 3 shows the correlations between latent factors and family cohesion as an observed variable. Academic failure correlated moderately with less parental education (*r* = −0.44, *p* < 0.001). In addition, child-to-parent violence is related to less family cohesion (*r* = −0.54, *p* < 0.001) and more positive family discipline (*r* = 0.56, *p* < 0.001) with moderate correlations. It is noteworthy the lack of relation between academic failure and child-to-parent violence (*r* = 0.11, *p* = 0.11).

**Table 3:** **Correlations between latent variables and one observed variable**.

**Latent variables**	**1**	**2**	**3**	**4**
1. Academic failure	**–**			
2. Child-to-parent violence	0.11	**–**		
3. Parental education level	−0.44^**^	−0.03	**–**	
4. Positive family discipline	0.16^*^	0.56^**^	0.04	**–**
**Observed variable**				
5. Family cohesion	−0.23^**^	−0.54^**^	0.14^*^	−0.20^**^

** p < 0.001;

* *p < 0.01*.

### Structural model

**Figure 1:**
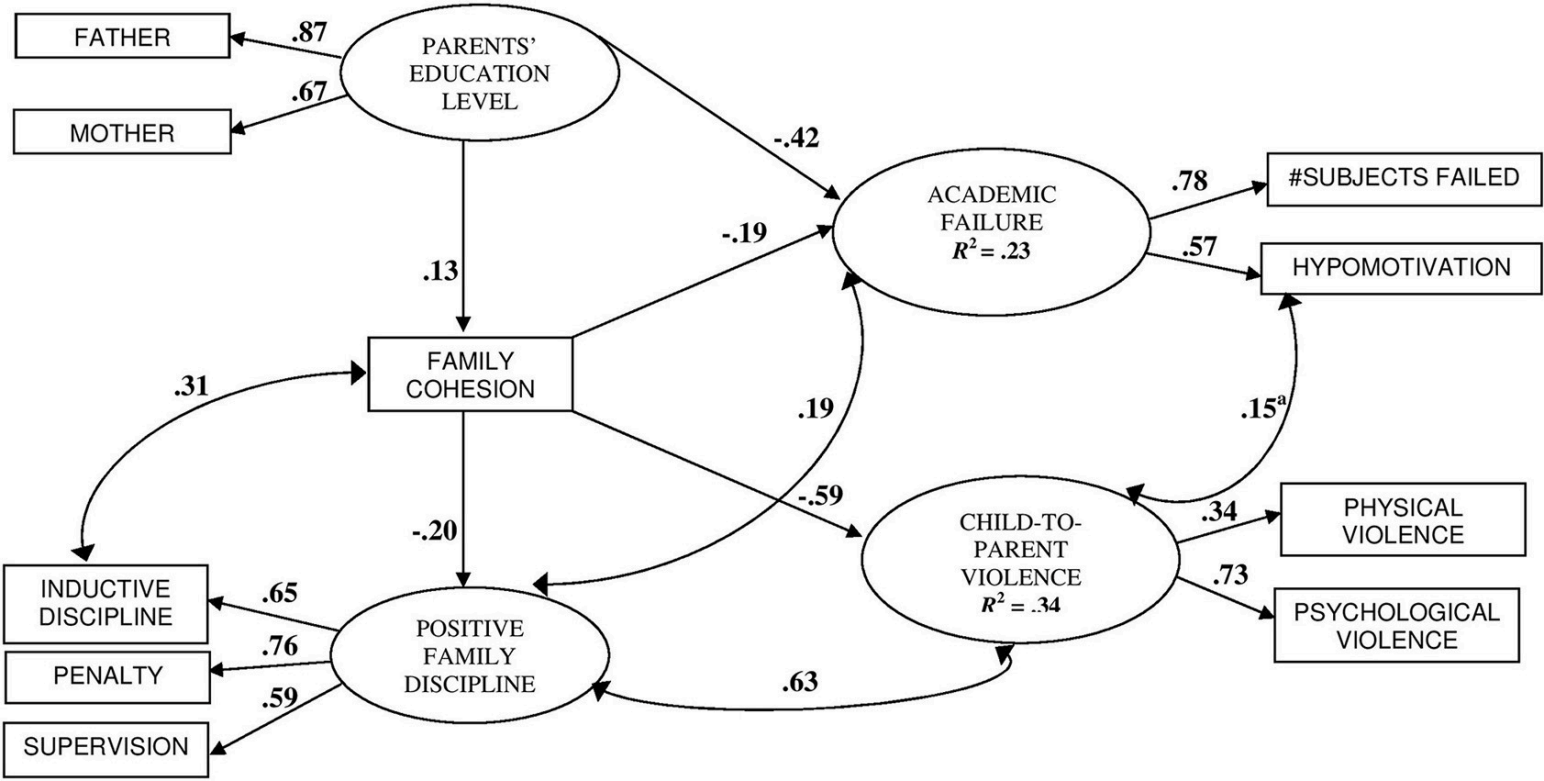
**Structural model predicting academic failure and child-to-parent violence**. Goodness of fit: *N* = 584; *ML* $\chi_{\left(27\right)}^{2}$ = 60.68; *CFI* = 0.98; *NNFI* = 0.96; *IFI* = 0.98; *RMSEA* = 0.038. All factor loadings, regression coefficients and correlations are significant, *p* < 0.01, except^a^ = *p* < 0.05.

The structural model was acceptable, *Y-B ML* χ^2^_(27, **N** = 584)_ = 60.68, *CFI* = 0.98, *NNFI* = 0.96, *IFI* = 0.98, *RMSEA* = 0.038, and this model accounted for 23% of the variance in academic failure and 34% of child-to-parent violence. All factor loadings were highly significant (*p* < 0.001). This structural equation model is presented in Figure 1, with standardized coefficients and associated probability.

On the one hand, family cohesion inversely predicted the use of Positive Family Discipline (β = −0.20, *p* < 0.001), School Failure (β = −0.19, *p* < 0.001), and Child-to-Parent Violence, (β = −0.59, *p* < 0.001). Parental education showed direct effects on Academic Failure (β = −0.42, *p* < 0.001), and indirect effects through family cohesion on Child-to-Parent Violence (β = −0.027, *p* < 0.01). In addition, school hypomotivation was related to child-to-parent violence (β = 0.15, *p* < 0.05). On the other hand, Positive Family Discipline was associated significantly with higher level of Academic Failure (*r* = 0.19, *p* < 0.01), and more Child-to-Parent Violence (*r* = 0.63, *p* < 0.001). An alternative model based on reverse causality between family cohesion and Academic Failure was also acceptable, *Y-B ML* χ^2^_(27, **N** = 584)_ = 67.72, *CFI* = 0.97, *NNFI* = 0.95, *IFI* = 0.97, *RMSEA* = 0.043, *R*^2^ = 0.20. The fit of this model was a little bit worse than previous one, and the model accounted less explained variance in academic failure. In this model family cohesion was predicted significantly by less Academic Failure (β = −0.20, *p* < 0.01). At the same time, family cohesion presented mediational effects between Academic Failure and Child-to-Parent Violence (β = 0.11, *p* < 0.01).

## Discussion

The objective of this study was to examine the contribution of several family variables on academic failure and child-to-parent violence in adolescents through a SEM model. As predicted, parental education level and family cohesion had some direct effects on academic failure. Previous findings indicated that higher parental education level was associated with less academic failure (e.g., Jensen, 2007; Li-Grining, 2007). The results of this study are fully consistent with those of previous research insofar as they show that students perform better academically the higher the levels of family economy and education, because they have more family resources, these being significant predictors of school industriousness (Shavit and Blossfeld, 1993; Caro et al., 2009; Córdoba et al., 2011). This may be due to greater parental involvement in their children's education, as previous work has shown a significant association between parental involvement and school performance of children (González-Pineda and Núñez, 2005).

It is also well-known that positive family relationships predict children's academic performance (Spera, 2005) and children's adjustment (Moreno et al., 2009; Jaureguizar et al., 2013). According to Blondal and Adalbjarnardottir (2009), the quality of the parent-child relationship seems to better predict the likelihood of the child staying in school than do specific parental actions aimed directly at the child's education. Moreover, the results of the present study highlight the higher association between parental education level (compared to professional category or unemployed status) and academic failure.

As hypothesized, positive family discipline strategies were associated with academic failure and child-to-parent violence. It seems that parents may apply discipline strategies to try and solve the academic achievement problems of their children. Taking into account Table 3, control or coercive strategies even moderate-level ones (penalty and supervision), are related to higher levels of academic failure, while positive family relationships predict greater academic success. These results are consistent with the conclusions of some studies in the Spanish context which indicate that adolescents from “indulgent” families (low control and high affect) present the same or better psychological adjustment than adolescents from authoritative families (high control and high affect; Musitu and García, 2004; García and García, 2010). Spera (2005), in his review, found that authoritative parenting style is often related to higher levels of academic performance of children, although this result is not consistent across cultures, ethnicity, or socioeconomic status. It should be highlighted that culture plays a meditational effect in the association between parenting styles and school achievement of adolescents. On the other hand, in this study inductive discipline was not associated with less academic failure. In a previous study by Ibabe (2015), inductive discipline was not associated with less child-to-parent violence, whereas coercive strategies did predict such behavior. These results are not contradictory with the importance of family discipline strategies as control strategies in order to have a positive influence on general indicators of adjustment and competence in adolescents, such as self-esteem or life satisfaction (Steinberg and Silk, 2002).

With regard to the third hypothesis, the results of this study confirm that children from one-parent-families or step-families show higher rates of academic failure than those living in nuclear families. This result is in line with the findings of previous studies (e.g., Córdoba et al., 2011). It was also observed that parents' divorce is related to poor academic self-concept (Orgilés et al., 2012). Fernández et al. (2010) suggest that marital separation processes are associated with at least four factors of family life which in turn can be associated with poorer school results: (1) lack of one parent, (2) parents with traumatic experiences, (3) economic impoverishment linked to dissolution of the marital relationship, and (4) other sources of instability in family life. Parental separation is associated to low psychosocial well-being of children, and it could explain their lower academic performance (Potter, 2010). However, if family give their children sufficient support or caring in the educational context they could have academic success.

The fourth hypothesis was partially fulfilled respect to direct effect of school failure on child-to-parent violence, because the academic failure predicted physical child-to-parent violence. This result is consistent with the study by Ibabe (2014) in which school maladjustment was not correlated with psychological child-to-parent violence, otherwise that physical, emotional and financial violence against parents. The positive association between physical child-to-parent violence and academic failure could be explained because both are indicators of children maladjustment. Adolescents who behave disruptively at home exhibit also behavior problems in school context. For example, disruptive behavior at school is an important predictor for aggression by adolescents toward their mothers (Pagani et al., 2004). In a study by Ibabe et al. (2013) was confirmed the importance of family environment over school environment for antisocial and violent behavior in adolescents. Taking into account the magnitude of relationship found in different studies, it seems that school maladjustment rather than academic failure is associated with child-to-parent violence.

As it was hypothesized school failure had indirect effects on child-to-parent violence through family cohesion. In this study school failure predicted low family cohesion and at the same time low family cohesion was a predictor of child-to-parent violence. This means that school failure has indirect effects on child-to-parent violence. On the one hand, school performance can be a social stressor for families producing family conflict and low cohesion, because it is associated with the parents' expectations about the children's scholastic achievements and prospective aspirations (Hurrelmann et al., 1988). On the other hand, family cohesion was a significant protective factor of child-to-parent violence. It is well-known that when there is negative environment in families, as indicated by having family conflict, marital violence, or parent-to-child violence, will be more probably that children use violence against parents, as indicated some previous studies (Gámez-Guadix and Calvete, 2012; Jaureguizar et al., 2013; Ibabe, 2015; Contreras and Cano, 2016).

In summary, this study highlights the effects of family context on academic achievement in adolescence, with parental education level to the fore. When students have difficulties to reach the teaching goals and they are characterized by behavioral problems as child-to-parent violence. Implications for professional in schools would be the focus on courses for parents in order to generate appropriate learning environments at home, and on the improvement of parent-child relationships. When parental education level is low, educational programs should be designed at the community level to target students with a view to improving their habits relating to studying, eating, and leisure activities (Córdoba et al., 2011).

The most important limitation of this study is that, as is the case in cross-sectional studies, the direction of causality cannot be established. Moreover, there is a risk that participants' motivation to respond may be affected by social desirability, so that they may overestimate their parental education level or their own academic achievement and study motivation, as socially acceptable features. Finally, all variables were based on children's self-reports.

## Author contributions

II makes substantial contributions to conception and design of the work, and/or acquisition of data, and/or analysis and interpretation of data; Drafting the work or revising it critically for important intellectual content; Final approval of the version to be published; and Agreement to be accountable for all aspects of the work in ensuring that questions related to the accuracy or integrity of any part of the work are appropriately investigated and resolved.

### Conflict of interest statement

The author declares that the research was conducted in the absence of any commercial or financial relationships that could be construed as a potential conflict of interest.
